# Diagnosis of human herpes virus 1 and 2 (HHV-1 and HHV-2): use of a synthetic standard curve for absolute quantification by real time polymerase chain reaction

**DOI:** 10.1590/0074-02760160354

**Published:** 2017-02-16

**Authors:** Lyana Rodrigues Pinto Lima, Amanda Perse da Silva, Jonas Schmidt-Chanasit, Vanessa Salete de Paula

**Affiliations:** 1 Fundação Oswaldo Cruz-Fiocruz, Instituto Oswaldo Cruz, Laboratório de Desenvolvimento Tecnológico em Virologia, Rio de Janeiro, RJ, Brasil; 2 Bernhard Nocht Institute for Tropical Medicine, Diagnostic Virology Laboratory, Hamburg, Germany

**Keywords:** real time PCR, synthetic curve, herpes

## Abstract

The use of quantitative real time polymerase chain reaction (qPCR) for herpesvirus detection has improved the sensitivity and specificity of diagnosis, as it is able to detect shedding episodes in the absence of clinical lesions and diagnose clinical specimens that have low viral loads. With an aim to improve the detection and quantification of herpesvirus by qPCR, synthetic standard curves for human herpesvirus 1 and 2 (HHV-1 and HHV-2) targeting regions gD and gG, respectively, were designed and evaluated. The results show that synthetic curves can replace DNA standard curves in diagnostic herpes qPCR.

The human herpesvirus or herpes simplex virus (HHV or HSV) is a neurotropic virus that has two distinct serotypes, human herpesvirus 1 and 2 (HHV-1 and HHV-2). Although both viruses are closely related, they contain sufficient differences to enable type identification (Nicoll et al. 2012). Historically, HHV-1 was considered the main cause of orolabial lesions, and HHV-2 was most commonly associated with genital infections. However, HHV-1 is increasingly being detected in genital lesions, and HHV-2 in orolabial lesions (Bhattarakosol et al. 2005). HHV is highly prevalent in many countries, and HHV infection is a global public health problem (Looker et al. 2015). Cell culture is the classic method used in the laboratory to diagnose herpes infection; however, this method is time-consuming and has low sensitivity (Curtin et al. 2013).

The ability to detect nucleic acid has had a major impact on clinical virology diagnosis (Niesters 2002). Polymerase chain reaction (PCR) is widely used in HHV research, and among the available PCR methods, quantitative real time PCR (qPCR) has the advantages of speed and quantification. In qPCR, the viral load is measured as the copy number per cell or percentage of total DNA by using a standard curve. A standard curve is generated by qPCR using a dilution series of a DNA template, which is commonly generated from plasmid DNA or DNA oligonucleotides (Tourinho et al. 2015). The advantage of using DNA oligonucleotides is that only the nucleotide sequence needs to be synthesized. For laboratories that do not have enough space or funding for molecular cloning, synthetic curves could be used as an alternative for quantification (Tourinho et al. 2015). Based on these advantages, the aim of this study was to evaluate the potential use of a DNA synthetic oligo as a standard for HHV-1 and HHV-2 quantification.

HPLC-purified oligonucleotides representing an 84-bp sequence of glycoprotein D in HHV-1 and a 91-bp sequence of glycoprotein G in HHV-2 were synthesized (Table I). The DNA oligonucleotides were diluted in DNase/RNase-free distilled water to 100 pmol/µL, which is approximately 10^13^ DNA molecules/µL, according to Avogadro’s number (Tourinho et al. 2015). These templates were evaluated by qPCR using the primers and probes previously described by Weidmann et al. (2008). Each qPCR contained 12.5 µL of TaqMan® Universal Master Mix (Applied Biosystems, Foster City, CA, USA), 2 µM of each primer, 1.5 µM of probe, and DNase- and RNase-free H_2_O. The cycling conditions were as follows: 95ºC for 10 min (initial denaturation and polymerase activation) followed by 40 cycles of denaturation at 95ºC for 15 s, and annealing and extension at 60ºC for 60 s. In this study, the standard curves were replaced with a ten-fold dilution series (10^1^-10^20^) of Ultramer® oligonucleotides. After determining the dilution range for each synthetic curve, they were assayed along with DNA for use as a standard curve to quantify virus in copies/µL.

**TABLE I:** Human herpesvirus-1 (HHV-1) and Human herpesvirus-2 (HHV-2) synthetic (oligo) standard curves

Synthetic ultramer	Sequence	Size (pb)	Amount of oligo (nmoles)
HHV-1	5’-TTCGTCTCGTAAAATGGCCCCTCCCGTATGGTTCGTCGGTGTGGTCGGTATGGATGCGTCGATAGTGTCACACGGCCGCTGATA-3’	91	4.4
HHV-2	5’-ATGCTATCTACCCACACACAGACCCACGTACGATCTGGTATCTGGTACTCGAATGTCTCCGCGCATGCAGGGAAGCATTTACGAGAGCGCTGATC-3	84	2.8

Each dilution of the HHV-1 and HHV-2 synthetic curves was compared to the DNA standard curve from virus preparations (quantification range was 10^0^-10^8^ copies/µL). First, the cycle thresholds (CT) of the viral DNA and synthetic oligo curves for both viruses (HHV-1 and HHV-2) were compared. For HHV-1, the CT differences between the DNA and synthetic curves were 0.29-1.28, and for HHV-2, the differences were 0.18-1.1. The amplification efficiency values were E = 97.2% (slope = -3.432, R^2^ = 0.996), with a detection limit of 10^2^ copies/µL for the HHV-1 synthetic curves, and E = 98.4% (slope = -3.285, R^2^ = 0.998), with a detection limit of 10^1^ copies/µL for the HHV-2 synthetic curves (Figure). The standard curve included seven dilution points, and the quantification ranged from 10^2^ to 10^8^ copies/µL for HHV-1 and from 10^1^ to 10^7^ copies/µL for HHV-2.

After evaluating the synthetic standard curves, they were used to quantify HHV-1 and HHV-2 levels in 33 clinical samples by real time qPCR as described above. The 33 samples, which were previously tested by qualitative PCR and with known viral loads, included 15 clinical samples of HHV-1 (two from cell culture, five lesions, five serum samples, and three saliva samples) and 18 clinical samples of HHV-2 (two from cell culture, three lesions, eight serum samples, two saliva samples, and three cervical scrapes). The assay results did not exhibit any discordance by quantitative or qualitative PCR. Furthermore, when DNA samples (each with 20 ng of DNA) from different sources were tested with both standard curves, the absolute quantification did not exceed one log (Table II).

**TABLE II:** Comparison of the absolute quantification of human herpesvirus-positive (HHV) samples using synthetic and DNA standard curves

HHV-1			HHV-2		
	
Samples	DNA curve (copies/uL)	Synthetic curve (copies/uL)	Samples	DNA curve (copies/uL)	Synthetic curve (copies/uL)
Lesion 1	1.3x10^6^	2.5x10^6^	Cervical scrape 1	7.8x10^5^	6.7x10^5^
Lesion 2	1.5x10^4^	2.9x10^4^	Cervical scrape 2	1.9x10^4^	1.0x10^4^
Lesion 3	5.7x 10^6^	7.1x10^6^	Cervical scrape 3	7.9 x 10^4^	6.8x10^4^
Serum 1	3.2x10^2^	4.3x10^2^	Serum 1	2.1.x10^1^	5.3x10^1^
Serum 2	1.0 x10^0^	1.9x10^0^	Serum 2	2.9x10^2^	3.5x10^2^
Serum 3	4.6x10^1^	3.8x10^1^	Serum 3	5.9x10^0^	6.7x10^0^
Serum 4	2.7x10^1^	1,9x10^1^	Serum 4	6.8x10^2^	7.6x10^2^
Serum 5	2.7x10^2^	1.8x10^2^	Serum 5	1.7x10^1^	4.7x10^1^
Cell culture 1	8.9x10^7^	9.5x10^7^	Serum 6	5.2 x10^1^	8.3x10^1^
Cell culture 2	6.6x10^7^	7.8x10^7^	Serum 7	3.4x10^2^	4.3x10^2^
Saliva 1	7.4x10^3^	8.7x10^3^	Serum 8	6.6x10^2^	7.4x10^2^
Saliva 2	3.2x10^3^	4.4x10^3^	Cell culture 1	9.4x10^6^	7.5x10^6^
Saliva 3	5.9x10^3^	7.1x10^3^	Cell culture 2	3.2x10^7^	2.4x10^7^
-	-	-	Saliva 1	2.9x10^2^	3.8x10^2^
-	-	-	Saliva 2	9.1x10^2^	9.8x10^2^
-	-	-	Lesion 1	7.5x10^5^	6.3x10^5^
-	-	-	Lesion 2	3.3x10^6^	1.5x10^6^
-	-	-	Lesion 3	2.2x10^5^	1.0x10^5^

Other authors have described improvements in the sensitivity and specificity of HHV diagnosis, and they were able to detect shedding episodes in the absence of clinical lesions (Aliabadi et al. 2015, da Silva et al. 2015, Bohórquez et al. 2016, Phipps et al. 2016, Ramchandani et al. 2016) and diagnose clinical specimens that had a viral load lower than lesion swabs, such as cerebral fluid, plasma (Tang et al. 2010), saliva, and cervical scrapes, as was demonstrated in the present study.

The use of this synthetic curve is currently limited to scientific research laboratories. However, previous studies have shown that oligonucleotides are good alternatives for quantification methods (Bowers & Dhar 2011, Tourinho et al. 2015). This study demonstrated that synthetic curves could be used as alternative standard curves for HHV diagnosis, since they showed similar results when compared to viral DNA curves.

**Figure:**
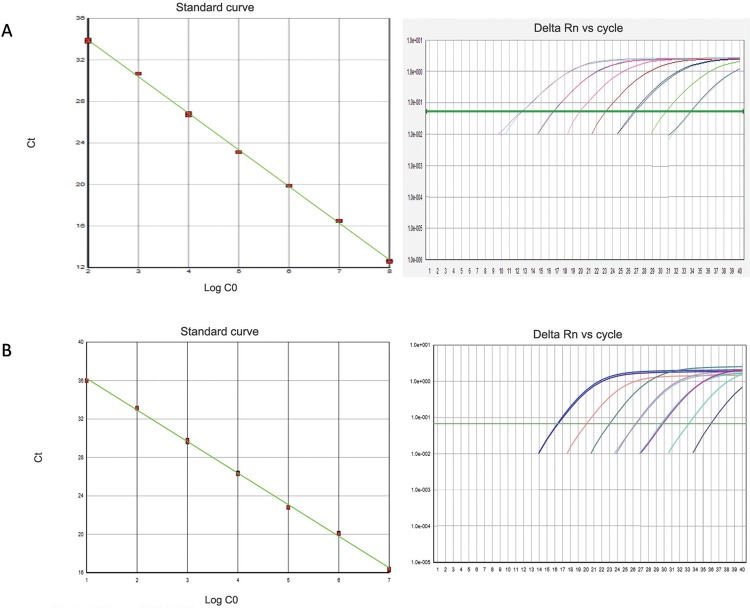
Amplification plots on a log scale and standard curves. (A) Human herpesvirus-1 (HHV-1); (B) human herpesvirus-2 (HHV-2).
